# Electro-vibrational stimulation results in improved speech perception in noise for cochlear implant users with bilateral residual hearing

**DOI:** 10.1038/s41598-023-38468-0

**Published:** 2023-07-12

**Authors:** Alexander Geerardyn, Katleen De Voecht, Jan Wouters, Nicolas Verhaert

**Affiliations:** 1 ExpORL, Department of Neurosciences, KU Leuven, Leuven, Belgium; 2 Department of Neurosciences, Leuven Brain Institute, KU Leuven, Leuven, Belgium; 3 Department of Otorhinolaryngology–Head and Neck Surgery, University Hospitals Leuven, Herestraat 49, 3000 Leuven, Belgium

**Keywords:** Transduction, Cochlea

## Abstract

A cochlear implant is a neuroprosthetic device that can restore speech perception for people with severe to profound hearing loss. Because of recent evolutions, a growing number of people with a cochlear implant have useful residual acoustic hearing. While combined electro-acoustic stimulation has been shown to improve speech perception for this group of people, some studies report limited adoption rates. Here, we present electro-vibrational stimulation as an alternative combined stimulation strategy that similarly targets the full cochlear reserve. This novel strategy combines the electrical stimulation by the cochlear implant with low-frequency bone conduction stimulation. In a first evaluation of electro-vibrational stimulation, speech perception in noise was assessed in 9 subjects with a CI and symmetrical residual hearing. We demonstrate a statistically significant and clinically relevant improvement for speech perception in noise of 1.9 dB signal-to-noise ratio. This effect was observed with a first prototype that provides vibrational stimulation to both ears with limited transcranial attenuation. Future integration of electro-vibrational stimulation into one single implantable device could ultimately allow cochlear implant users to benefit from their low-frequency residual hearing without the need for an additional insert earphone.

## Introduction

Worldwide more than 430 million people are suffering from disabling hearing loss with the need for rehabilitation^1^. For people with moderately severe to profound levels of hearing loss, speech perception can be restored by a cochlear implant (CI)^2^. A CI is a surgically implantable device that replaces the function of the hearing organ by converting the sound waves of speech and music into electrical pulses. These electrical pulses are then conveyed to the brain via the auditory nerve.

CIs are considered among the most successful implantable medical devices and can provide excellent speech perception in quiet^2,3^. In situations with multiple competing talkers or background noise, however, speech perception remains much more difficult^4,5^. For example, even CI users with excellent word recognition in quiet need at least 5–10 dB better signal-to-noise ratio (SNR) to reach the same speech perception as normal hearing listeners^5,6^. These difficulties with speech perception in background noise have been attributed to the lower spectral resolution with a CI resulting from, among other factors, the limited numbers of electrodes and current spread inside the cochlea^4,7^.

As a result of the recently evolving CI indication criteria, there is a growing number of CI candidates with useful residual acoustic hearing at the time of the implantation^8^. In the majority of cases, the residual hearing is located in the low-frequency range from 125 to 1000 Hz. Moreover, due to advances in electrode designs and surgical techniques, long-term preservation of this residual hearing after implantation has been shown to be possible in about 70% of patients^9^. In the future, integration of new technologies such as real-time monitoring during CI insertion may further increase hearing preservation rates^10^.

Interestingly, combining low-frequency acoustic amplification with the electrical stimulation by the CI, so-called electro-acoustic stimulation (EAS), has consistently been shown to improve speech perception, especially in background noise^11–13^. The benefits of this combination have been attributed to a better representation of voicing and manner of articulation, allowing the user to narrow down the potential word candidates in the lexicon^14,15^. Furthermore, in background noise, the voicing cues can provide acoustic landmarks for when to listen to the target speaker^14,15^. Nevertheless, despite the benefits and high adoption rates of EAS observed in clinical trials^12,13,16^, a recent survey in a population with a low EAS adoption rate identified that about half of the users reject EAS for non-audiological reasons, with the need for an extra insert earphone being the most cited^17^.

In the present study, we explore the potential of *Electro-Vibrational Stimulation* (EVS) as an alternative combined stimulation strategy for the first time. EVS combines the electrical stimulation by the CI with vibrational stimulation provided by a bone conduction (BC) actuator. With EVS, we aim to improve speech perception in the growing group of CI users with residual hearing, while overcoming the limitations of EAS.

Nowadays, BC devices are widely used in clinical practice^18^. In contrast to hearing aids that amplify sounds via a speaker in the ear canal, BC actuators convert the sound into mechanical vibrations. These vibrations are then transferred directly to the inner ear, largely bypassing the outer and middle ear. Recently, implantable actuators have been developed^19,20^. By integrating implantable actuator technology into the CI housing, in the future, EVS could be delivered by a single implantable device. This would allow CI users to benefit from the high spectral and temporal resolution of their low-frequency residual hearing without the need for an additional insert earphone.

The aim of this study was to assess speech perception in noise with a non-invasive EVS prototype for the first time. In a within-subject repeated measures design, the speech reception threshold (SRT) with EVS was compared to the SRT with the CI alone (E-only) in CI users with symmetrical residual low-frequency hearing. We hypothesized that speech perception in noise would improve by adding low-frequency vibrational stimulation to the electrical stimulation of the CI.

## Results

**Figure 1:**
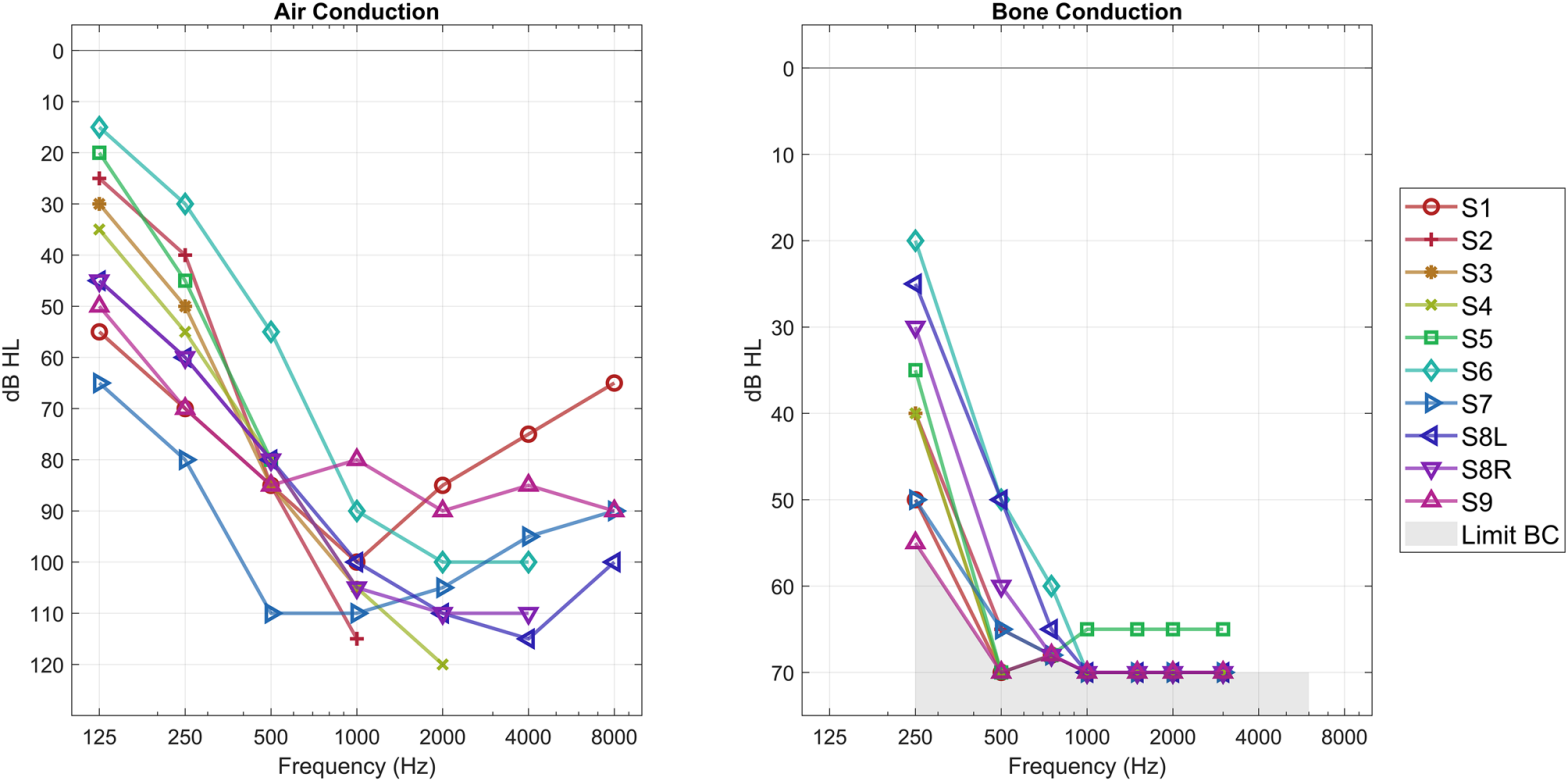
**Pure tone audiometric thresholds.** Left Panel: Pure tone air conduction thresholds on the ipsilateral side of the cochlear implant. Right Panel: Pure tone bone conduction thresholds (unmasked) obtained with transcutaneous stimulation at with the commercial BC device, so-called BC Direct thresholds. The shaded grey area indicates the limit of the output of the BC in the commercial fitting software.

In 7/10 ears the pattern of hearing loss was continuously down-sloping from low to high frequencies, while in 3/10 the most profound hearing loss was located in the mid frequencies (Fig. 1, Left Panel). The mean low-frequency pure tone average with air conduction (LFPTA-AC; 125 Hz, 250 Hz, 500 Hz, 1000 Hz) at the side of the implant was 68 dB HL. The mean asymmetry in LFPTA-AC between the implanted and non-implanted ear was 5 dB HL. Full pure tone audiogram data are available in the supplementary materials.

The BC direct thresholds are plotted in Fig. 1—Right Panel together with the maximum power output (MPO) of the BC device. Except for subject 9, each of the participants had at least one frequency (250 Hz) with a BC threshold of at least 5 dB lower than the MPO. BC thresholds obtained with the Radioear B71/81 are available in the supplementary materials.

**Figure 2:**
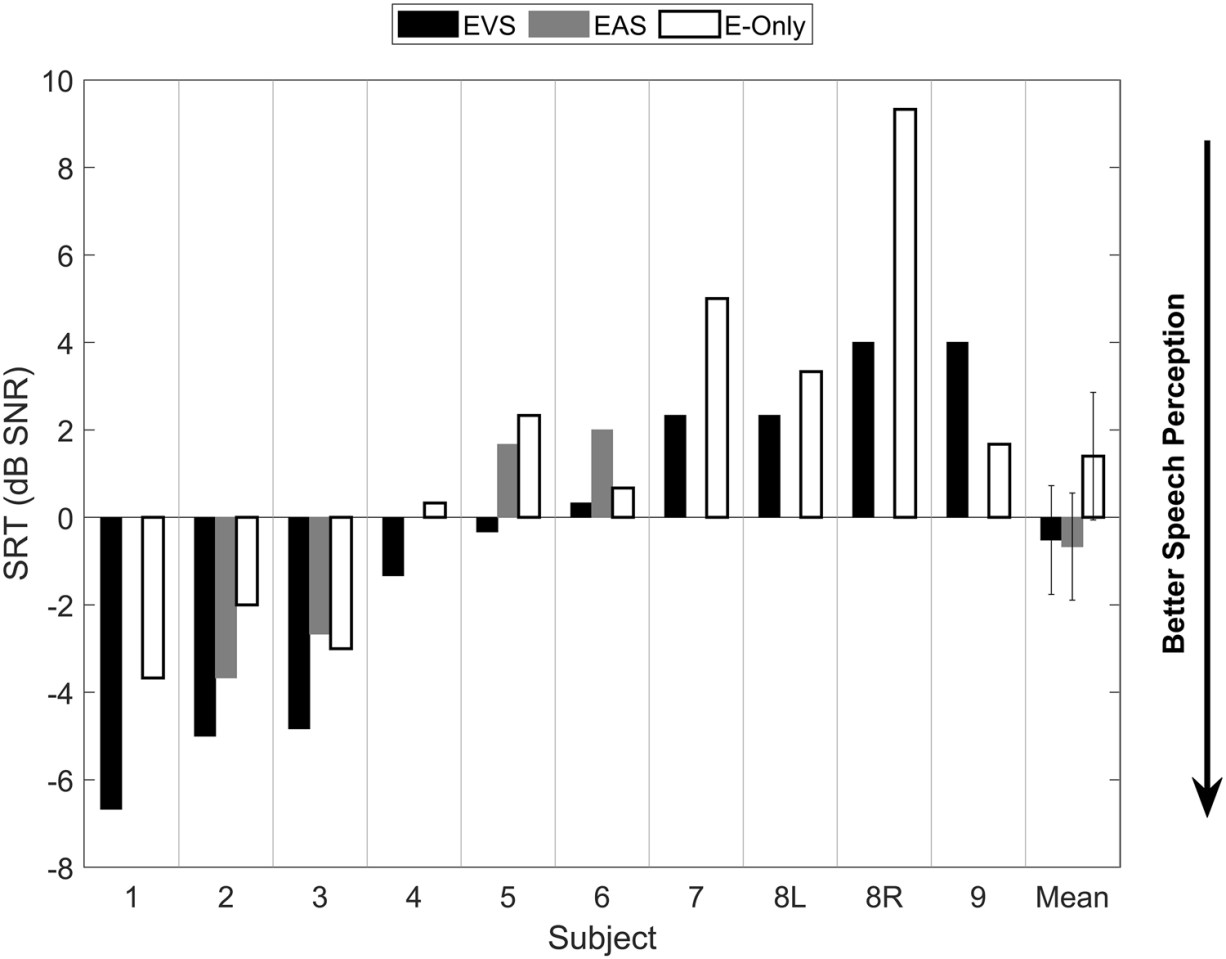
**Speech reception thresholds with different device conditions. **Lower scores indicate a better speech perception. Note: The mean SRTs for EVS, EAS and E-only are calculated on different sample sizes. SRT: Speech Reception Threshold. SNR: signal to noise ratio. Error bars indicate the standard error of the mean.

Individual SRTs in different conditions are plotted in Fig. 2. Speech perception in noise was significantly better with the EVS condition than with the E-only condition (*p* = 0.015). The mean (n = 10) SRT with the E-only condition was 1.4 dB SNR (SD: 4 dB SNR, Range: −3.7 to 9.3 dB SNR) compared to − 0.5 dB SNR (SD: 3.9 dB SNR, Range: −6.7 to 4.0 dB SNR) with the EVS condition. The mean EVS benefit was 1.9 dB SNR (SD: 2.0 dB SNR). On an individual level, each of the participants benefited from EVS compared to E-only, except subject 9.

In the subgroup with a restricted CI frequency spectrum (n = 3) the SRT with E only and EVS were on average − 1.4 and − 3.2 dB SNR, respectively. In the subgroup with a full CI frequency spectrum (n = 7) the SRT with E-only was 2.6 dB SNR compared to 0.6 dB SNR with EVS.

We observed no correlation between the degree of low-frequency residual hearing, measured as LFPTA-AC or low-frequency pure tone average with BC (LFPTA-BC), and SRTs with E-only, SRTs with EVS or EVS benefit (Pearson Correlation, p > 0.05; scatter plots are available in the supplementary materials).

In the subgroup of patients with EAS (n = 4) we observed a non-significant average improvement in speech perception with EAS over E-only—i.e., EAS effect—of 0.17 dB SNR. The average improvement with EVS compared to EAS in the same subgroup was 1.8 dB SNR.

## Discussion

We have shown for the first time that EVS can improve speech perception in steady-state noise compared to E-only. In a population of experienced CI users with symmetrical low-frequency hearing the SRT improved on average 1.9 dB SNR with EVS compared to E-only. The observed effect size is considered clinically significant and in line with the improvement of 2 dB SNR observed with EAS over E-only in a large multi-center study^13^. In contrast to EAS, however, this benefit was achieved by providing BC stimulation at the level of the skull, without the need for insert earphones.

Also, in the subgroup of participants with EAS (n = 4), we observed a better speech perception with EVS compared to EAS. However, this result should be interpreted with caution because of the small sample size and the relatively smaller EAS effect (i.e., SRT E-only -SRT EAS) of 0.17 dB SNR in this subgroup group compared to what is typically observed in larger EAS studies^12,13^. Moreover, given the bilateral BC stimulation of EVS (see paragraph on binaural stimulation below), a comparison with unilateral EAS should be interpreted with caution. Individually, 8 out of the 9 subjects benefited from the addition of vibrational stimulation. Individual effect sizes ranged from 0.3 to 5.3 dB SNR. Only subject 9 did not experience an EVS benefit and performed even worse compared to E-only (− 2.3 dB SNR). This lack of improvement in subject 9 may be due to multiple factors. One potential factor that stands out is the level of the low-frequency BC thresholds in this particular subject. At each of the frequencies between 250 and 1000 Hz, the BC thresholds matched or exceeded the MPO of the BC device used in this study. As a consequence, the effective dynamic range of the BC device—i.e., the difference between the BC threshold and the loudest hearing level that can be obtained with the device^21^—was close to zero for this subject.

While in each of the other participants, the effective dynamic range of the BC device was > 0 for at least one frequency, the typically desired 30–35 dB effective dynamic range was obtained only for two subjects (S6; S8L) at 250 Hz^21^. There are two primary reasons for the limited effective dynamic range of the BC device used in this study. First, we repurposed a commercially available BC device. While this device was the most powerful commercially available BC device at the time of this study, it has not been designed for the specific purpose of the present study to generate high-output low-frequency stimulation. Moreover, because of the exploratory nature of the study including human participants, we opted for a non-invasive setup via transcutaneous stimulation. This stimulation method is associated with skin attenuation of the BC vibrations, albeit limited in the frequency range < 1000 kHz^22^. Nevertheless, despite this limited effective dynamic range, we did observe a significant benefit of EVS on speech perception in noise. The effective dynamic range of EVS could further increase with a dedicated BC actuator designed for higher output in the low frequencies and direct coupling to the skull bone. Based on the results from previous studies^23,24^ that showed improved speech perception with higher output power BC devices, this could result in even larger effect sizes with EVS vs E-only. However, it remains unclear how the findings from these studies showing the benefit of an increased broadband output would translate to higher low-frequency stimulation at the electro-vibrational cross-over frequency.

**Figure 3:**
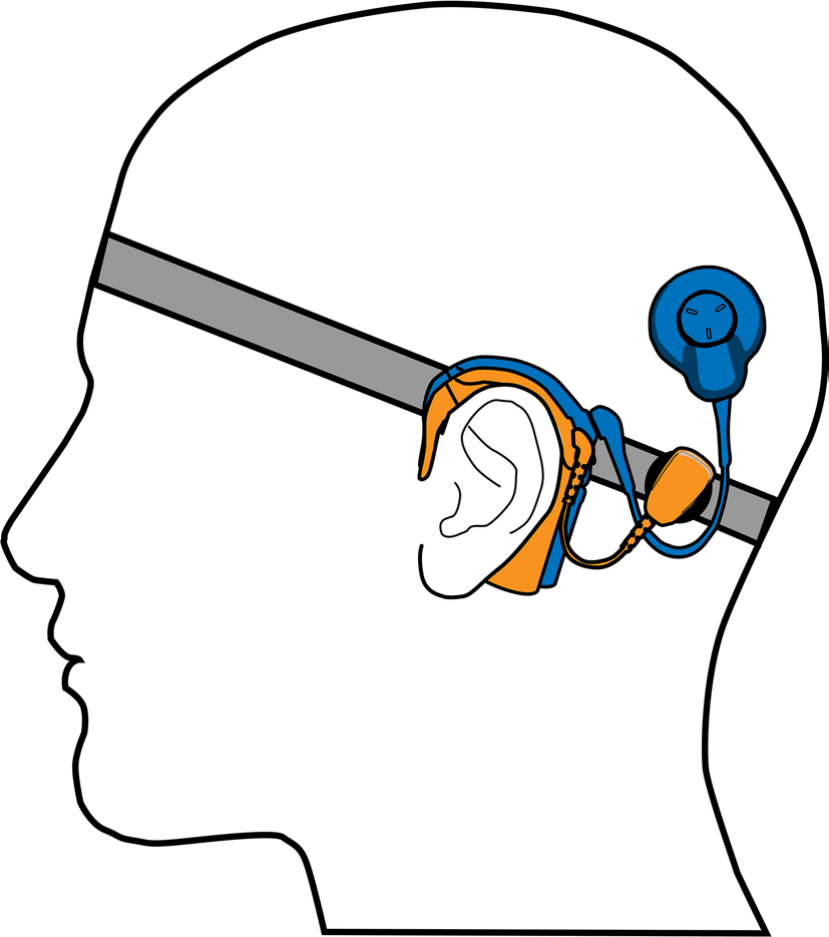
**Schematic of electro-vibrational stimulation as used during the experiments.** Illustration of how the combination of the cochlear implant (blue) and bone conduction device (orange) are worn on the headband (grey) by the participants during the experiments.

For the subjects that did benefit from EVS with the current setup (Fig. 3), we hypothesize the observed improvement in speech perception in noise is mainly facilitated by the same mechanisms as observed with EAS. The better speech perception in noise with EVS vs E-only could be attributed to the superior representation of voicing and manner of articulation in the low-frequency vibrational information, narrowing down the potential word candidates in the lexicon. Moreover, in background noise, the more pronounced acoustic landmarks can provide a cue to the user when to listen to the target speaker^14,15^.

Furthermore, two mechanisms specific to EVS could contribute to improved speech perception in noise. First, EVS could potentially provide haptic cues. It is known that, especially for the low-frequency range, the MPO levels of the present BC actuator are well above the vibrotactile thresholds at the mastoid^25^. Previous studies showed that low-frequency tactile stimulation at the wrist or fingertip can improve speech perception in noise when combined with the electrical stimulation by the CI^26,27^. Though this electro-haptic effect was not specifically assessed in the current experiment, it may have contributed to the improved speech perception in noise. However, the study by Fletcher et al. only showed a significant average increase in word recognition of 8.3% with haptic stimulation after a training window of 20 min which is longer than the total duration of EVS use in the present study experiments^26^. Based on the data obtained with the current patient population, we cannot differentiate the exact contribution of the electro-haptic effect. In future studies, this isolated effect can be measured by applying the same methodology of EVS in participants with a CI and the absence of residual hearing in both ears.

A second mechanism specific to EVS is the bilateral stimulation with little transcranial attenuation, especially in the low frequencies^28^. Since all the participants in the present study had symmetrical low-frequency residual hearing, this could result in binaural summation. This phenomenon of binaural summation has been shown to improve speech perception in steady-state noise up to 1.3 dB SNR in normal-hearing listeners^29,30^. The impact of binaural summation in bone conduction stimulation, however, is less clear.

The present within-subject repeated measures design has the advantage of limiting the inter-subject variability that is typically observed with CI users. Yet, this also implies that subjects are tested in device conditions that differ from their usual rehabilitation. Therefore, to further limit the number of modifications from the rehabilitation strategy of a single participant, we chose to keep the CI fitting map unaltered.

This unaltered CI fitting could negatively impact speech perception E-only condition in EAS users. In 3 out of the 4 EAS users included in this study, the frequency range covered by the CI was restricted at the low-frequency end. As a result, these subjects miss out on a small part—i.e., 188–313 Hz or 188–438 Hz—of the frequency spectrum in the E-only condition. The restricted frequency spectrum in the E-only condition could negatively impact speech perception and result in an artificially high EVS effect (SRT EVS—SRT E-only). While we cannot exclude the effect of the altered frequency map completely, posthoc analyses comparing the SRT with E-only, SRT with EVS, and EVS benefit in the group with a full CI frequency spectrum (n = 7) to those with a restricted frequency spectrum (n = 3) support the idea that EVS benefit is substantial and not solely based on an unfavorable E-only condition. While the results should be interpreted with caution given the small sample sizes, we did even observe opposite trends with better SRTs in E-only condition and lower EVS benefit in the group with the restricted E-only frequency spectrum.

**Figure 4:**
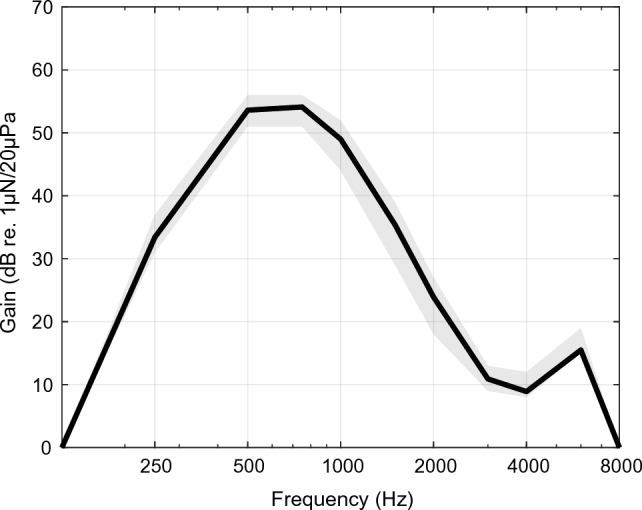
**Fitting curve bone conduction device.** The black line shows the average fitting. The shaded area shows the range between different participants.

The fitting parameters of the vibrational component in EVS were identical for all subjects (Fig. 4). Because of the variation in residual BC hearing thresholds, the resulting frequency spectrum allocated to the vibrational component varied between subjects. In combination with the (near-)full spectrum CI fitting, this resulted in a variable degree of spectral overlap or redundancy of the frequency spectra allocated to the electrical and vibrational component in all subjects. Previous studies in EAS indicate that limiting the spectral overlap or redundancy may further improve speech perception in noise^31,32^. While it is unclear if these findings can be generalized to EVS, we hypothesize that individualization of the allocation of the frequency spectra could further increase EVS benefit.

In the present study, stimuli were presented in free field without the use of ear plugs or ear defenders to block the AC pathway. Some of the subjects with excellent residual hearing and thresholds of up to 15 dB HL may have benefited from unamplified low-frequency acoustic cues in the E-only condition, therefore resulting in a smaller observed EVS effect than would be the case if the air conduction pathway was blocked. However, since no condition with occlusion of the AC pathway was included in this study, we do not have data to support this hypothesis. In any respect, the reported EVS effect sizes in the present study really show the potential impact of adding a vibrational component in CI users with residual hearing that currently use only their CI (E-only).

Finally, it should be noted that the experiments concerned an acute in-hospital evaluation and that the speech perception tests with EVS occurred after only a short accommodation time of 5–10 min. Based on the improvements in speech perception over time observed in previous studies with E-only or electro-tactile stimulation, even larger effect sizes may be expected if the subjects have a longer adaptation time to the new EVS condition before testing^26,33,34^.

One of the main limitations of the present study is the size and heterogeneity of the study population. Nevertheless, each individual participant except the one participant with the poorest BC thresholds has been shown to benefit from EVS. The current sample does not allow further analysis of the impact of different electrode types, fitting maps, or the use of a contralateral hearing aid. Future studies should aim to recruit a larger homogenous sample to further explore these effects.

In summary, our result show for the first time that EVS can improve speech perception in noise for CI users with symmetrical residual low-frequency hearing and could be an alternative to EAS. Even larger effect sizes can be expected with an individualized frequency spectrum allocation and a longer adaptation time. Future integration of EVS into one single implantable device could ultimately allow CI users to benefit from their low-frequency residual hearing without the need for an additional insert earphone.

## Methods

### Participants

Nine adults with a CI, 6 females and 3 males, were prospectively recruited from the University Hospitals Leuven. The following inclusion criteria were applied: (1) Low-frequency residual hearing—i.e., at least one frequency between 125 and 1000 Hz in the ipsilateral ear with a pure tone audiometric threshold ≤ 65 dB HL; (2) Minimum of 6 months of experience with the CI; (3) Stable fitting of the CI over the last month before testing; (4) Native Dutch speaker. This study was approved by the Ethics Committee of the UZ Leuven (S65654) and performed in accordance with the Declaration of Helsinki. Informed consent was obtained before the start of the experiments.

Participant demographics are displayed in Table 1. Except for subject 8 all participants in this study had been implanted with a single CI in one ear. The duration of implantation (i.e., CI experience) ranged from 7 to 176 months. Except for subject 7 who was implanted with an Advanced Bionics (AB, Valencia, CA USA) device, all participants used a Cochlear™ (Sydney, Australia) Nucleus Processor. Of the 8 subjects with a single ipsilateral CI, 6 were wearing a hearing aid in the contralateral ear (i.e., bimodal stimulation). While all participants had some degree of residual hearing in the ipsilateral CI ear, only 4/9 were actively using EAS. The frequency spectrum covered by the CI via electrical stimulation differed between the implant manufacturers and between EAS users and non-EAS users. The majority of the CIs were fitted to cover the spectrum from 188 to 7398 Hz electrically. The single AB implant had slightly different cut-off frequencies (250–8700 Hz). In 3/4 of the EAS users, the CI frequency spectrum was restricted with the lower limit frequency shifted upwards from 188 Hz to up to 438.

**Table 1:** Participant characteristics.

Subject	Sex	Age	Side	Implant type	Months since implantation	Current rehabilitation		Frequency spectrum CI	
						Left ear	Right ear	Lower limit (Hz)	Upper limit (Hz)
1	F	24	R	Cochlear Nucleus CI 622	7	HA	E-only	188	7398
2	F	60	R	Cochlear Nucleus CI 522	55	HA	EAS	438	7938
3	F	21	L	Cochlear Nucleus CI 622	21	EAS	HA	313	7398
4	F	25	R	Cochlear Nucleus CI 522	89	–	E-only	188	7398
5	F	65	R	Cochlear Nucleus CI 622	7	–	EAS	188	7398
6	M	63	R	Cochlear Nucleus CI 622	7	HA	EAS	313	7398
7	M	50	R	AB HIFocus SlimJ (18)	36	HA	E-only	250	8700
8	M	18	R	Cochlear Nucleus CI 422	94	E-only	E-only	188	7398
			L	Cochlear Nucleus CI24RE	176	E-only	E-only	188	7398
9	F	65	R	Cochlear Nucleus CI 522	45	HA	E-only	188	7398

*F* female, *M* male, *CI* cochlear implant, *EAS* electro-acoustic stimulation, *HA* hearing aid.

### Audiometric and otologic evaluation

At the start of the experiment, air conduction thresholds (125, 250, 500, 1000, 2000, 4000, 8000 Hz) were obtained for both ears. Only participants with a difference in LFPTA-AC between the two ears of < 15 dB HL were included in this study. BC thresholds were obtained with the RadioEar B71/81. Otomicroscopy and tympanometry were performed to rule out middle ear effusion or external ear canal occlusion as potential causes of conductive hearing loss.

### Device fitting

In this study speech perception was assessed in up to three device conditions: E-only (CI only), EVS (CI + BC stimulation at the ipsilateral mastoid), and EAS (CI + acoustical stimulation by a HA in the ipsilateral ear). The EAS condition was only tested if used by the participant in daily life. No changes were made to the fitting of the acoustical component of the EAS or the acoustical HAs in the contralateral ear. In this first preliminary assessment of EVS, a non-invasive stimulation combination is used. The previously implanted CI provided the electrical stimulation. The vibrational component was delivered by a transcutaneous BC device. More specifically, we used the *Cochlear™ Baha® 5 SuperPower*, the most powerful commercially available BC in terms of MPO at the time of the study. To keep the BC device in place at the mastoid ipsilateral to the CI, the participants wore an elastic headband (Fig. 3). This non-invasive method is frequently used in young children with conductive hearing loss and for adults with conductive hearing loss or single-sided deafness before deciding on the definitive implantation of a bone screw^22^. Special attention was paid to the BC actuator not to interfere with the CI coil or implant body.

Pure tone BC thresholds (i.e., *BC Direct*) were obtained with the transcutaneous BC device using the commercially available fitting software (*Baha® Fitting Software 5.4*) provided by the manufacturer. These thresholds are unmasked and, because of the limited attenuation of BC stimulation, represent the lowest threshold of the ipsilateral and contralateral ear combined. The LFPTA-BC was calculated based on thresholds at 250, 500, 750, and 1000 Hz. The output of the BC actuator was fitted similarly for each participant with maximum gain at frequencies below 1 kHz and minimal output at higher frequencies (Fig. 4). Standard signal pre-processing algorithms including automatic directionality and noise suppression were consistently active on the commercially available BC actuator and unaltered between subjects or conditions.

The CI fitting map was unaltered. The experiments were performed with the fitting map used by the participant at home. The frequency spectrum covered by the CI is displayed in Table 1.

The loudness between different device conditions was assessed in a subjective manner by presenting a 60 dB SPL stimulus (ISTS noise^35^) and asking the participant for a score on a 7-levels scale as used for the assessment of T and C levels in CI fitting. This loudness assessment showed that the device conditions were perceived equally loud within one level difference on the 7-levels scale. Given the fixed BC output (Fig. 4) and unaltered E-only or EAS fitting map, no further frequency-specific loudness matching procedure was performed in the present study.

### Experimental Setup, Stimuli, and Procedure

The LIST (Leuven Intelligibility Sentence Test) sentences were used as speech stimuli. The LIST set is a Dutch speech material containing 35 equivalent lists of each 10 sentences uttered by a female speaker. Each sentence contains three to four keywords. The LIST speech material was specifically designed for testing people with severe hearing impairment and CI users^6^. The speech stimuli were presented in combination with steady-state speech weighted noise with a matching long-term-average speech spectrum.

The EVS condition was fitted at the beginning of the experimental procedure. The participants had a short period of 5–10 min to get accustomed to the new rehabilitation strategy before the start of the measurements.

The measurements were performed in a double-walled soundproof room. The stimuli were presented from a PC using APEX software^36^, routed via an RME Hammerfall DSP Multiface II sound card to a single speaker (Genelec 8020B) placed at a distance of 1.35 m in front of the participant at 0°.

Speech perception was assessed in a within-subjects repeated measurements design. Between different tests, only the device condition (E-only, EVS, or EAS) differed. In one experimental session with the same participant, up to three device conditions (e.g., EVS, E-only, EAS) were tested. During the experimental session, the participants did not use a contralateral device. In this acute experimental setup, there was only a short interval of about 5–10 min to get accustomed to the next device condition. The order of the device conditions was randomized between participants. Experiments with one participant were performed during a single session. The research design was unmasked to both participants and researchers.

In each specific test, SRT was determined via an adaptive procedure^6,37^. While the intensity of the speech stimulus was kept constant at 60 dB SPL, the SNR varied in a 1-up 1-down procedure by changes in the intensity of the masker in steps of 2 dB SPL. The SRT was calculated as the mean of the SNRs of the last 6 trials. The initial SNR at the beginning of the experiment (− 5, 0, or 5 dB SNR) was set at a level about 5 dB SNR lower than the SRT measured with E-only during previous testing at the clinic, resulting in a speech perception level close to 0%. The first sentence was repeated at increasing SNRs in steps of 2 dB SNR until correct. Each test with a specific device condition was repeated at least twice (test–retest). If the difference between the test and retest was > 2 dB, an additional retest was performed. The final SRT was calculated by averaging the two lowest (i.e., best) SRTs.

In none of the conditions ear plugs or ear defenders were used.

### Statistical analysis

Statistical testing was performed with SPSS version 28.0.1.1. We compared the within-subject effect of the device condition (i.e., EVSor E-only) on SRTs with a paired t-test (two-sided). The Kolmogorov–Smirnov test with Lilliefors modification was used to assess normality. Correlations were assessed with Pearson correlation. Significance levels were set at 0.05.

EVS and EAS benefits were defined as the difference in SRT between EVS and E-only condition or EAS and E-only condition, respectively.

## Supplementary Information

Supplementary Information 1.

Supplementary Figure 1.

Supplementary Figure 2.

Supplementary Figure 3.

## Data Availability

All data analyzed during this study are included in the published article.
