# Supplementary figures and images for: Electro-vibrational stimulation results in improved speech perception in noise for cochlear implant users with bilateral residual hearing
Source: Sci Rep. 2023 Jul 12;13:11251. doi: 10.1038/s41598-023-38468-0 (PMC10338449; doi:10.1038/s41598-023-38468-0)

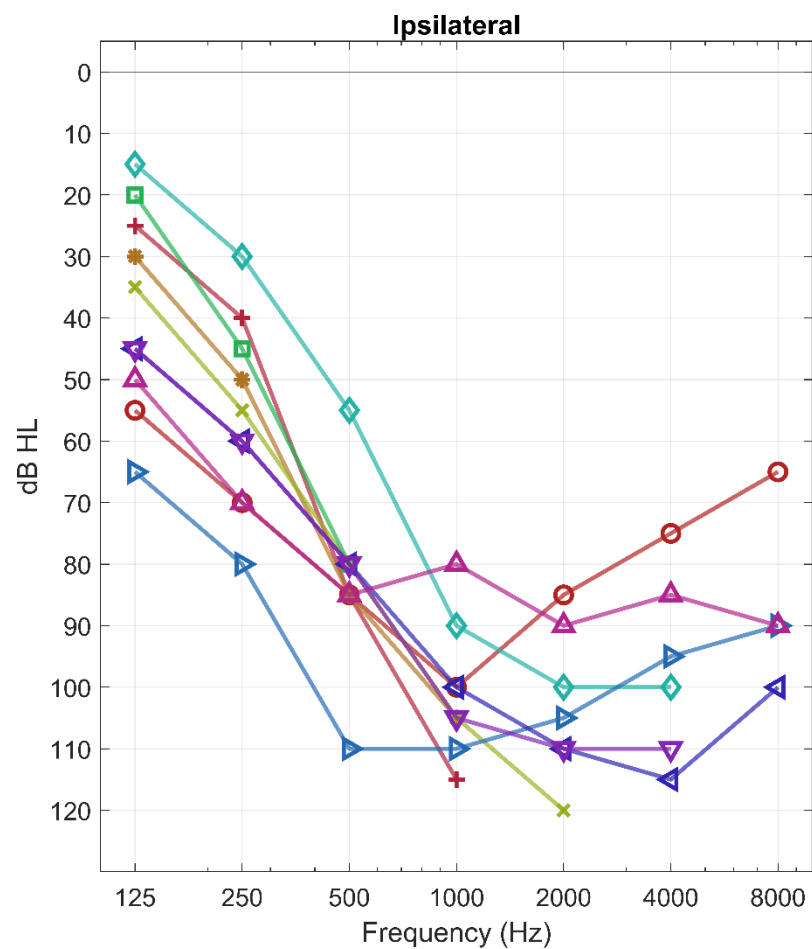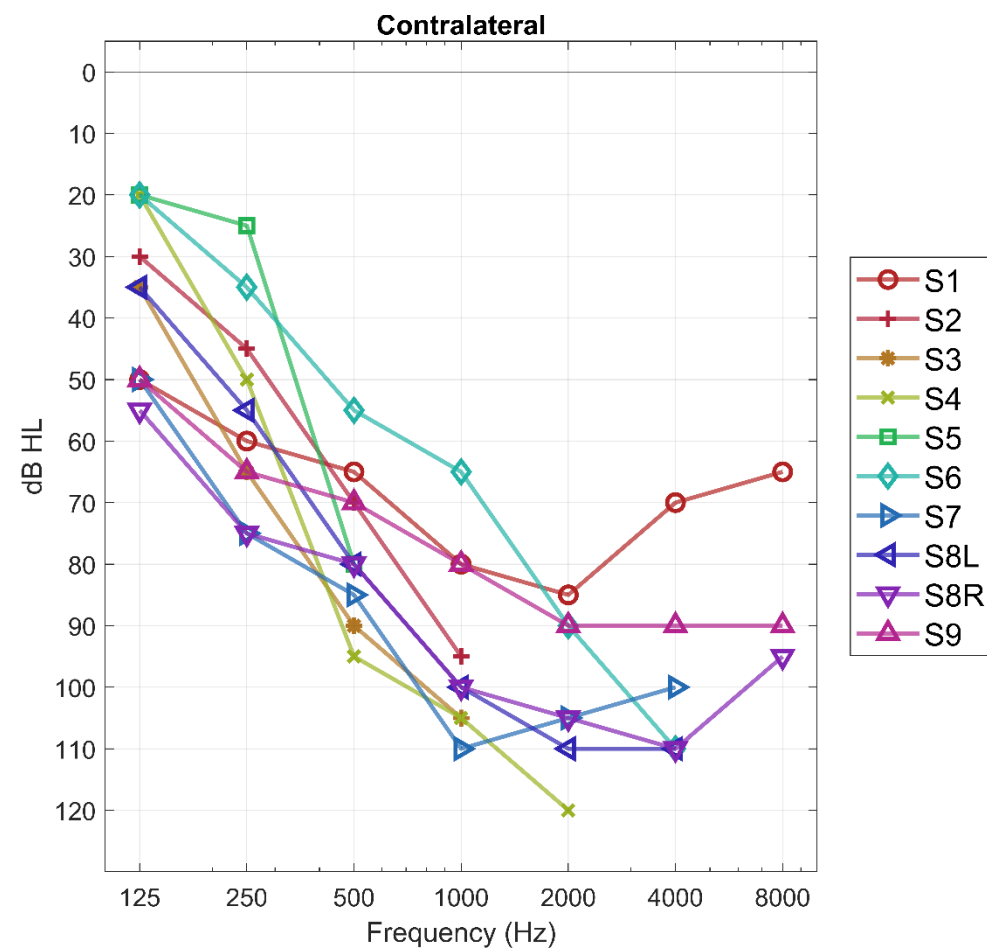

**Supplemental Figure 1:** Pure Tone Air Conduction Thresholds.

Supplement: Supplementary file 2 — Supplementary Figure 1. [file 41598_2023_38468_MOESM2_ESM.pdf]

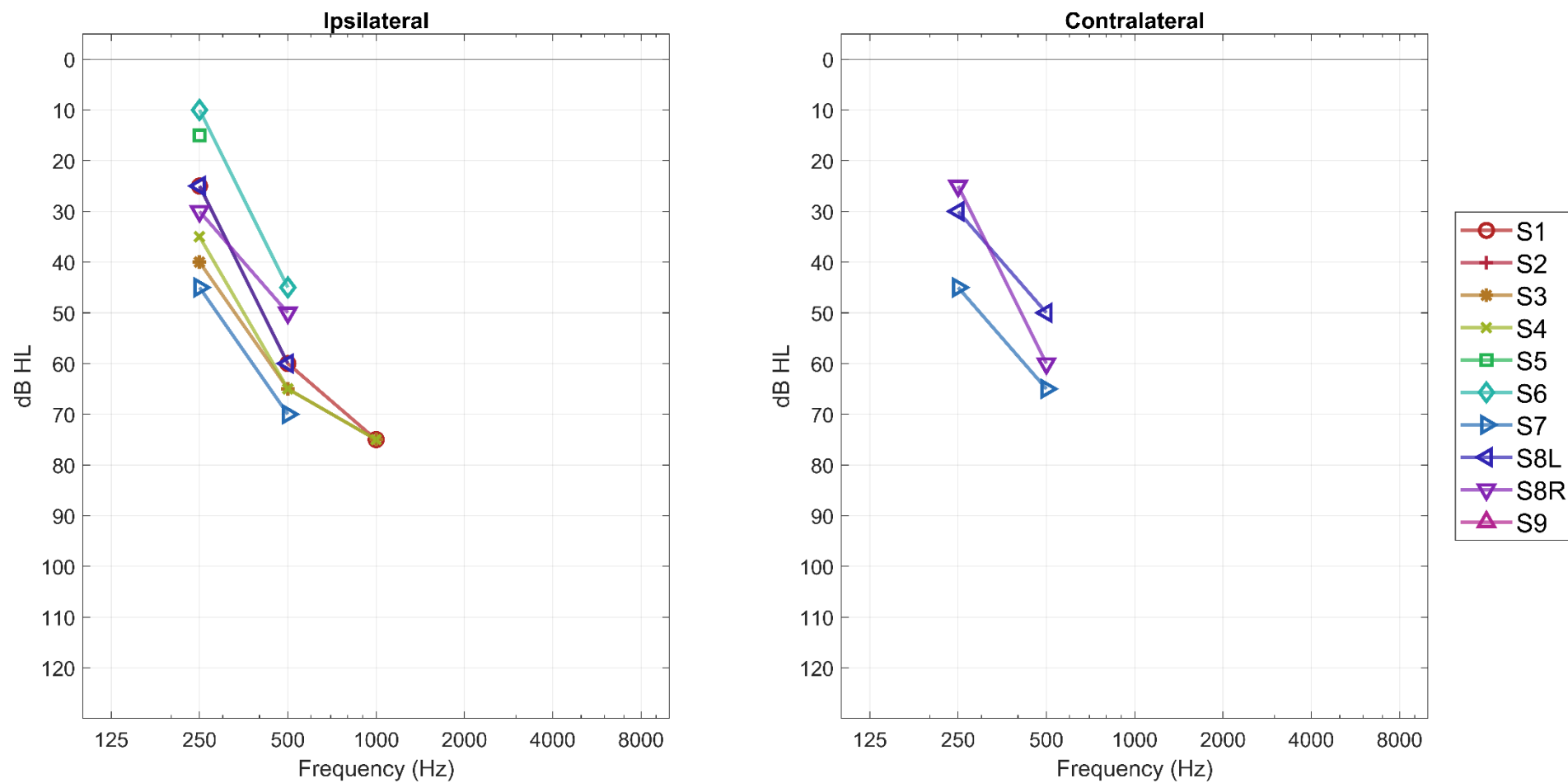

**Supplemental Figure 2:** Pure Tone Bone Conduction Thresholds (Masked).

Supplement: Supplementary file 3 — Supplementary Figure 2. [file 41598_2023_38468_MOESM3_ESM.pdf]
