# Supplementary material for: Electro-vibrational stimulation results in improved speech perception in noise for cochlear implant users with bilateral residual hearing
Source: Sci Rep. 2023 Jul 12;13:11251. doi: 10.1038/s41598-023-38468-0 (PMC10338449; doi:10.1038/s41598-023-38468-0)
Supplement: Supplementary file 4 — Supplementary Figure 3. [file 41598_2023_38468_MOESM4_ESM.pdf]

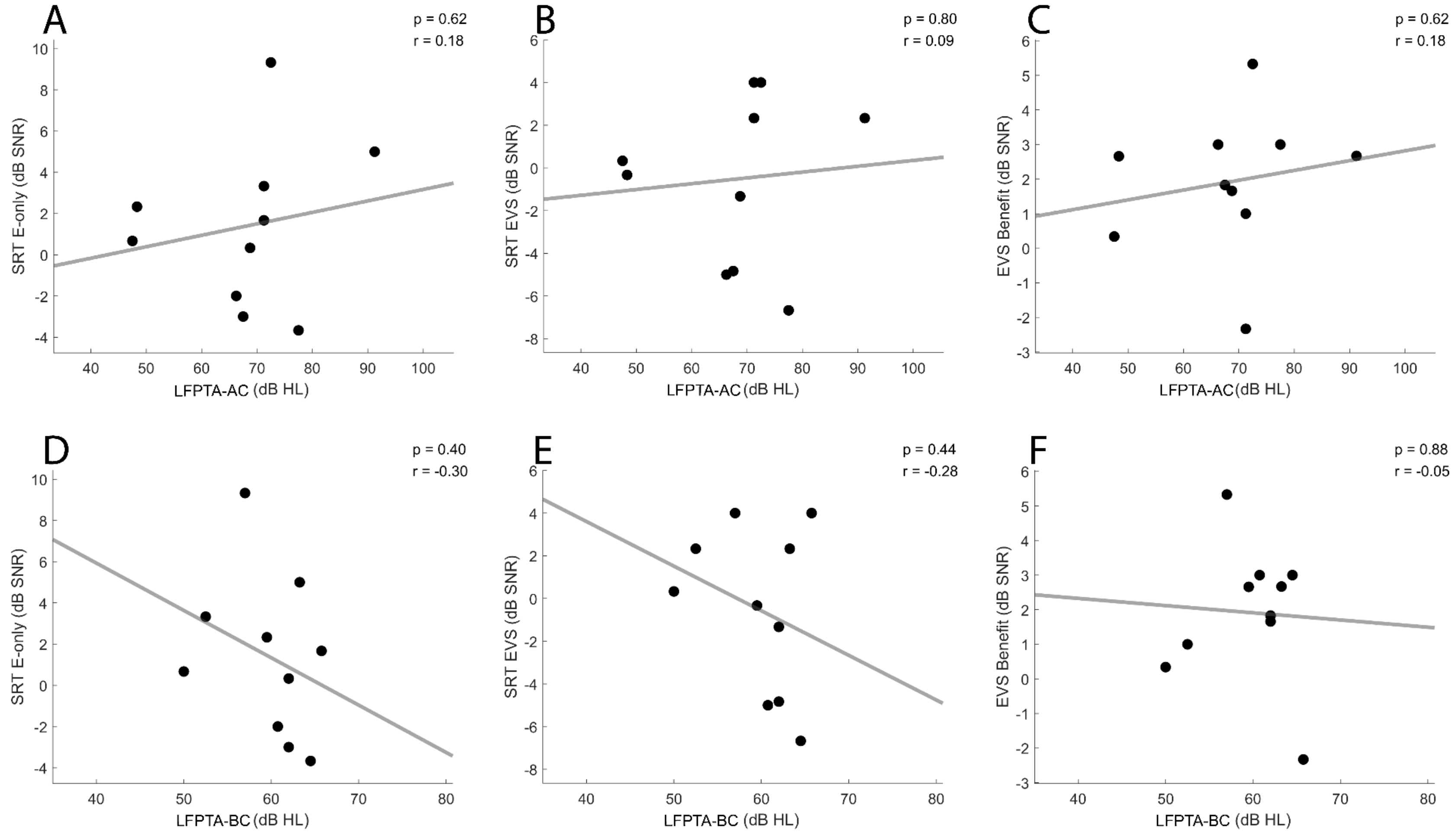

Supplementary Figure 3*: Scatter plots illustrating the correlations between the degree of residual hearing - either measured as low-frequency pure tone average with air conduction (LFPTA-AC, Panel A-C) or low-frequency pure tone average with bone conduction (LFPTA-BC, Panel D-F) - and speech reception threshold (SRT) with E-only, Electro-Vibrational Stimulation (EVS), or the difference between E-only and EVS. P and rho values are calculated according to Pearson correlation. The grey line indicates the least squares line.*
